# Initial experience of spatially fractionated lattice radiation therapy for palliative treatment of pediatric bulky tumors

**DOI:** 10.3389/fonc.2025.1648847

**Published:** 2025-10-27

**Authors:** Hualin Zhang, Kenneth Wong, Arthur Olch, Hye Ri Han, Brittney Chau, Lauren Lukas, Eric L. Chang

**Affiliations:** ^1^ Radiation Oncology Program, Children's Hospital Los Angeles, Los Angeles, CA, United States; ^2^ Radiation Oncology Department, University of Southern California, Los Angeles, CA, United States

**Keywords:** spatially fractionated radiation therapy, SFRT, pediatric patient, bulky tumor, heterogeneous dose

## Abstract

**Purpose:**

Spatially fractionated radiation therapy (SFRT) has demonstrated high clinical response rates with minimal toxicity in adult patients with bulky tumors, including radioresistant histologies. However, there is limited clinical data on SFRT in pediatric patients, and optimal techniques and dose regimens remain unclear. This study presents our single-institution experience with SFRT for the palliative treatment of bulky pediatric tumors.

**Methods and materials:**

A retrospective review was conducted on six pediatric patients with metastatic or unresectable bulky tumors treated with SFRT. SFRT was delivered using VMAT in the form of Lattice Radiation Therapy (LRT). SFRT fraction doses ranged from 10–15 Gy, with 7–26 high-dose vertices per treatment. Sequential conventional external beam radiation therapy was delivered in 6 courses (67%), and SFRT was used for re-irradiation in 3 courses (33%). A radiobiological modeling approach was employed to estimate treatment effects across varying cancer and normal cell radiosensitivity levels.

**Results:**

Six pediatric and young adult patients (median age: 10 years) received in total 9 SFRT courses. Tumor sites included the liver (4), abdomen (3), pelvis (1), and thorax (1), with a median gross tumor volume of 666 cc. Median follow-up was 1.7 months. Tumor volume reductions were observed in 67% of treated sites (mean reduction: 279.3 cc), with significant clinical improvements in symptoms (e.g., pain, gastrointestinal symptom relief) and no grade 3 or higher toxicities. Radiobiological modeling data indicates that SFRT improves the therapeutic ratio, particularly when cancer cells are radioresistant and surrounding normal tissues are radiosensitive. Increasing the number of high-dose cores may further enhance this ratio.

**Conclusions:**

SFRT seems to be a safe and effective palliative treatment for pediatric bulky tumor patients in our preliminary study. Radiobiologic modeling suggests that increasing the density of high-dose cores can enhance the therapeutic ratio, potentially reducing normal tissue toxicity.

## Introduction

Treatment options for large tumors are limited, as the dose from conventional radiation regimens may be insufficient for durable responses and stereotactic body radiation therapy (SBRT) is typically reserved for smaller tumors due to surrounding normal organ constraints. Spatially fractionated radiation therapy (SFRT) is an emerging technique that delivers high doses of radiation heterogeneously to sub-volumes called high-dose vertices (“peaks”), which are regularly interspersed among a low-dose background (“valley”) within the gross tumor volume (GTV) or clinical tumor volume (CTV) when there was large uncertainty in the actual border of the GTV, allowing safer treatment of large tumors near organs at risk (OARs). The enhanced tumoricidal effects of SFRT are attributed to high-dose peaks that enhance cancer cell kill and antigen release while low-dose valleys preserve the tumor’s immune microenvironment and vasculature (1, 2). These heterogeneous dose distributions are believed to promote bystander and abscopal immune effects, leading to greater-than-expected tumor responses. In the 1990s, SFRT was introduced through GRID therapy, which showed promising oncologic outcomes in the palliation of large or recurrent tumors (3–5). Since then, SFRT has continued to show high rates of clinical response with minimal toxicity in treating large-volume primary, metastatic, and refractory malignances, generally in adults. More recently, Lattice radiation therapy (LRT, or simply Lattice), a three-dimensional form of SFRT (5–7), has emerged. LRT takes advantage of the improved planning flexibility of modern treatment planning systems to achieve 3D heterogeneous dose modulation, offering more options for optimization than collimator-based GRID therapy. However, this variability of LRT can introduce inconsistencies that can complicate trial design and response assessment (8, 9). Therefore, LRT requires thorough evaluation, consensus, and standardization for specific disease sites under investigation.

Pilot studies of GRID and LRT SFRT have shown unexpectedly high tumor response rates with low toxicity in bulky primary and metastatic tumors in adult patients (3, 4, 10, 11). There have been multiple phase I or case studies evaluating SFRT using GRID or LRT in the adult population (2, 12–14). Site-specific studies have also examined SFRT in head and neck (15, 16), sarcoma (17–19), lung (20), and cervical cancers (21, 22). These studies report symptomatic response rates of 78-100% (2, 12) and radiologic response rates of 79-97%, with tumors exhibiting an approximately 50% reduction in size (12, 13, 23). Importantly, grade 3–4 toxicities associated with SFRT remain low (0-8%) (12, 14, 17). Early data also reports favorable survival outcomes in non-metastatic patients (16, 20, 21, 24), with long-term follow-up confirming an overall favorable toxicity profile. This suggests that SFRT may play a role in treating bulky primary, recurrent, and metastatic tumors, especially where conventional radiotherapy techniques are limited by normal tissue tolerance (25). Currently, single-institution clinical trials in Phase I or II are exploring both GRID and LRT techniques (26), and multi-institutional prospective clinical trials are under discussion (8, 26).

Although clinical data exists for SFRT in adult patients, pediatric SFRT data is lacking. To fill this knowledge gap and begin addressing the unique variables in pediatric patients, we report our retrospective analysis of palliative SFRT for bulky tumors in pediatric patients. Our study examines patient and treatment characteristics, treatment responses (symptom improvement, local control, and imaging response), toxicity, treatment planning, and dosimetric parameters.

## Methods

### Patient selection guidelines

This study was approved by the Institutional Review Board of Children's Hospital Los Angeles. A retrospective review was conducted between January 2022 and July 2024. All patients were 19 years of age or younger, with tumors which were unresponsive to systemic therapy and/or not amenable to surgery or chemotherapy. Prior radiation to the affected region was permitted.

### Tumor geometry and depth

The prescription dose was delivered to the tumor vertices, with less than 6% of the tumor volume receiving the prescribed dose. Each treatment used a Volumetric Modulated Arc Therapy (VMAT) plan with four arc fields, with a dose of 10 or 15 Gy prescribed to the tumor vertices at the 100% isodose line. The structure contours from the first SFRT course for each patient, along with the corresponding tumor sizes and locations within the 3D images, were shown in the **Supplementary Document** (**Appendix A**, **Supplementary Figure S1**).

### Lattice plan and dosimetric parameters

#### SFRT planning techniques

The CT simulation dataset, with 2 mm slice spacing and approximately 1 mm planar spatial resolution, was imported into Eclipse (Version 16.1, Varian Medical Systems, Palo Alto, CA). The gross tumor volume (GTV) was contoured, and a CTV expansion was created with an appropriate margin based on the tumor site and histology. Nearby OARs were also contoured. The VMAT-based Lattice technique was used to create SFRT plans for all patients. Following RSS GRID/Lattice Working Group guidelines (27), vertices (spheres) were created and populated within the GTV or planning target volume (PTV) or clinical tumor volume (CTV) when there was large uncertainty in the actual border of the GTV. The diameters of these vertices were typically 2 cm, depending on the size of the PTV, with the goal of placing as many vertices as possible while maintaining a high heterogeneous dose index D5/D95, the ratio of doses covering 5% and 95% of the target volume.

**Figure 1:**
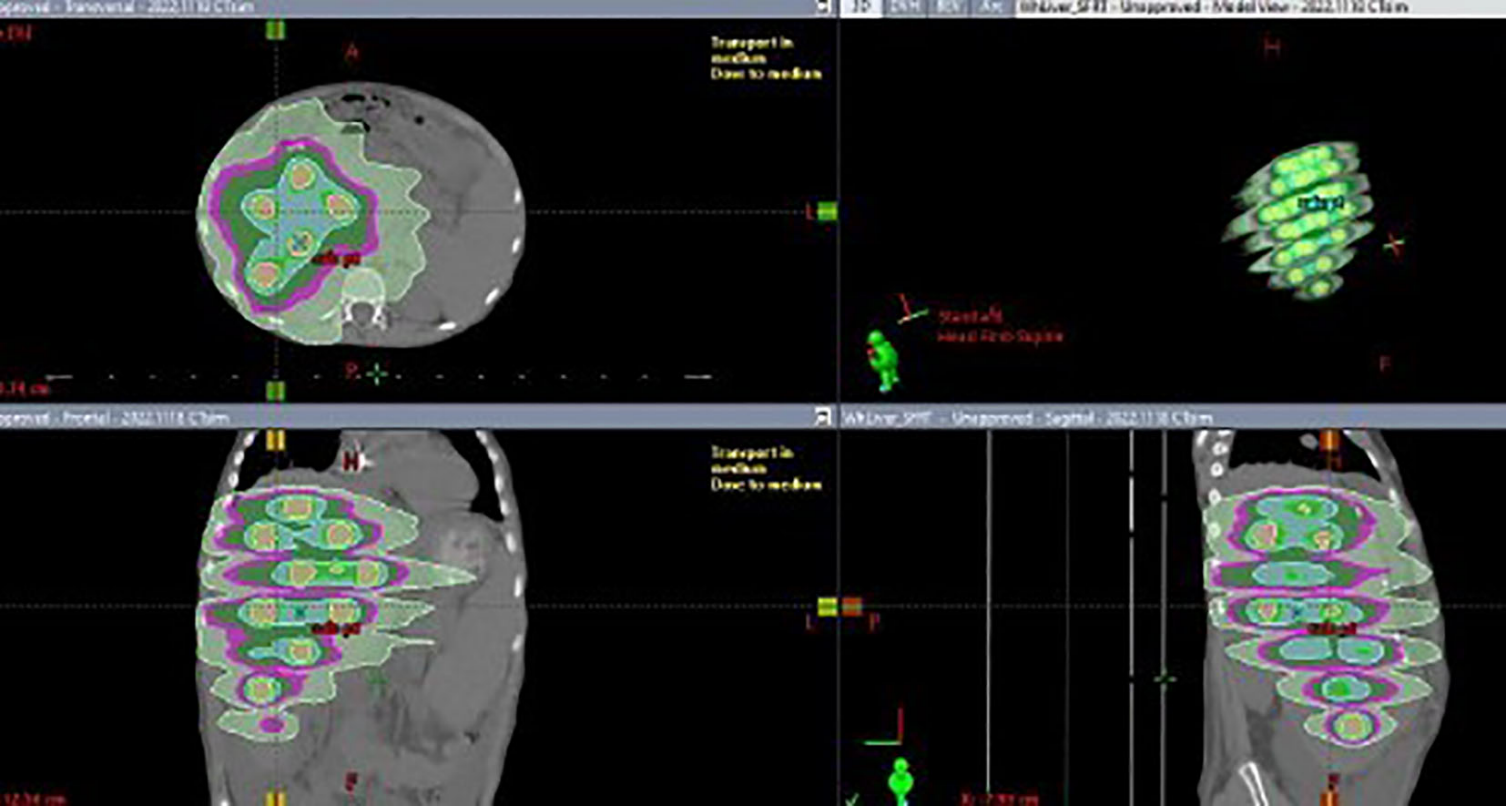
Dose distribution from an LRT plan for a bulky tumor in a pediatric patient.

The placement of these spheres adhered to the rule that the center-to-center distance between spheres be 4 cm, while maintaining a minimum distance of 1 cm from the edge of any sphere to the GTV border. The number, sizes and locations of these spheres were manually adjusted in 3D to approximately achieve this distribution. Once finalized, the union of all spheres was defined as the target structure. The optimizer was then run, and the lower objective dose was set to the prescribed dose for the union structure, while the upper objective was set slightly above this value. The normal tissue objective was set to forcefully constrain the isodose lines against the spherical PTVs, encouraging a rapid dose falloff to raise the peak-to-valley dose ratio or D5/D95. Dose calculations were performed using the Acuros XB algorithm (V16) with a 2 mm dose calculation resolution. Four 10 MV flattening filter-free VMAT arcs were used for each plan on a TrueBeam linear accelerator (Varian). **Figure 1** shows a typical SFRT plan’s dose distribution.

#### Dose metrics and radiobiology modeling estimation of Lattice plans

All SFRT dose metrics, including the doses covering 100%, 95%, 90%, 50%, 10% and 5% of the target volume (D100, D95, D90, D50, D10, D5, D10/D90, D5/D95), and the equivalent uniform dose (EUD) recommended by the RSS GLMF Working Group (28), were calculated for all plans. A newly introduced dose heterogeneity metrics, the high-dose core number density (HCND, number of vertices/GTV), was extracted as well.

The EUD was determined using a modified linear quadratic (MLQ) model, employing a consistent α/β ratio of 10 Gy specifically for cancer cells. Three different radiosensitivity scenarios were modeled based on surviving fractions (SF) at 2 Gy open field: radiosensitive (C1, SF(2Gy) = 0.3), semi-radiosensitive (C2, SF(2Gy) = 0.5), and radioresistant (C3, SF(2Gy) = 0.7) (29–32). To assess LRT’s impact on normal cells, tissue was classified as late responding (α/β = 3.1 Gy) with three radiosensitivity levels: SF(2Gy) = 0.3, 0.5, and 0.7, named N1, N2, and N3. Using the linear quadratic (LQ) model (33), α and β values for each cell type (cancer and normal tissue) were derived based on the given α/β ratio and assumed SF at 2Gy. A table presented in both the study for GRID therapy (32) and the **Supplementary Document** (**Appendix A**, **Table A**) of this study summarizes these values. The method, definitions and algorithms of radiobiologic modeling are described in detail in a study for GRID collimator-based SFRT (32).

Using the differential dose-volume histogram (d-DVH) curve of each plan, and assuming that cancer cells are uniformly interspersed in the target volume (an assumption that may not be true for late-stage tumors), the average survival fraction, $\bar{SF}\;$ for a given cancer cell radiosensitivity was calculated based on the MLQ equation and the sub-volumes of the tumor irradiated by corresponding doses. It is important to note that this averaging of cell survival assumes independent clonogens, excluding effects like bystander, abscopal, and cohort effects, which are hard to quantify and found more relevant to interspersed cancer cells than to normal tissues (34–36). In recent years, multiple studies have sought to quantify the advanced effects described in SFRT (37–40). However, the proposed algorithms are often too complex for routine clinical use or yield minimal contributions to improved clinical response. The integration of these advanced effects, along with other as-yet-unidentified factors, into a practical and validated algorithm continues to present a substantial challenge.

Using the average survival fraction, $\bar{SF}$ , an EUD for a given treatment and cancer cell scenario (i.e. radiosensitivity) can be calculated by solving the MLQ equation (31).

When the above cell survival estimation approach is applied to the interspersed normal cells, a metric of therapeutic ratio (TR) can be calculated (28).


(1) $$TR=\frac{SF_{Normal}\left(LRT\right)}{SF_{Normal}\left(EUD\right)}$$

In Equation 1, SF_Normal_ (LRT) and SF_Normal_(EUD) are the normal cell survival fractions respectively in LRT and EUD treatments. A TR > 1 implies that a greater number of normal cells survive in LRT than in EBRT at the same rate of cancer cell killing, indicating a therapeutic advantage of LRT over traditional EBRT for sparing normal cells (30, 31).

DVH curves summarize the 3D dose distribution within the tumor target volume, however, these curves do not capture the spatial fractionation information essential for SFRT. To address this, the high dose core number density (HCND) (41) was used to quantify spatial dose fractionation in SFRT. HCND is defined as the number (n) of peaks in GRID therapy or vertices in LRT, divided by the SFRT target volume (V) and multiplied by 100 cm^3^, as shown in the Equation 2,


(2) $$HCND=\frac{n}{V}*100\;cm^{3}$$

The co-relationship between the TR and HCND was examined in this study.

#### Treatment

VMAT plans were made using 10 MV flattening filter-free beams with various full or partial arcs. All plans met the prescribed dose constraints for both targets and OARs.

Prior to treatment, patient-specific plan QA was performed using an EPID-based QA system (SunCHECK™, Sun Nuclear, Florida). A standard SBRT quality assurance protocol was implemented, employing 2mm global dose and 2% distance to agreement criteria with 15% dose threshold. A minimum passing rate of 93% was attained across all cases. Treatment delivery followed standard VMAT protocols, beginning with image-guidance with cone beam CT (CBCT) to correct patient positioning and body posture before administering LRT.

## Results

### Patient demographics and treatment data

Tumor volumes ranged from 266 to 2306 cm^3^ (median: 666 cm^3^). Tumor equivalent sphere cross-sectional diameters at the center depth ranged from 8 to 16.4 cm (median: 11.2 cm), with the largest tumor dimension ranging from 9.4 to 22 cm (median: 15.5 cm). From December 2022 to July 2024, six patients received a total of nine SFRT courses. The median patient age was 10 years (range 11 months – 19 years). There were several histologies in various anatomic locations, such as neuroblastoma located in the thorax and abdomen, hepatocellular carcinoma (HCC) and hepatoblastoma in the liver, Wilms tumor in the abdomen, malignant rhabdoid tumor in the abdomen, and angiosarcoma in the pelvis. The median GTV was 666.1 cc (range 265.9-2306.5 cc). **Table 1** shows patient age and tumor characteristics. The SFRT dose ranged from 10–15 Gy in a single fraction (median: 15 Gy). Each lattice treatment utilized 7 to 26 high-dose vertices. SFRT was delivered before or after C-EBRT in six (67%) treatment courses. The C-EBRT dose ranged from 14 Gy in 4 fractions to 42.75 Gy in 15 fractions (median: 20 Gy in 4 fractions). SFRT was used for re-irradiation in three (33%) treatment courses.

**Table 1:** Patient tumor characteristics for lattice SFRT.

Case/course #	Age at time of treatment (years)	Histology, tumor site	Equivalent-sphere diameter (cm)	Largest dimension (cm)
1A	5	Neuroblastoma (PD)	13.7	17.7
1B	6	Neuroblastoma (PD)	9.5	11.5
2	17	Angiosarcoma (PD)	11.5	18.6
3	0.92	Rhabdoid tumor of the kidney (RD)	8.0	9.4
4A	10	Hepatocellular neoplasm NOS (RD)	16.4	22.0
4B	10	Hepatocellular neoplasm NOS (RD)	13.3	16.5
5	5	Wilms tumor, anaplastic (RD)	8.0	12.8
6A	18	Hepatocellular carcinoma, fibrolamellar (RD)	10.5	17.3
6B	19	Hepatocellular carcinoma, fibrolamellar (RD)	9.5	14.0
Mean	10.1		11.2	15.5
Median	10		10.5	16.5

Cases 1A and 1B represent a single patient treated with two courses of SFRT to two different areas. Cases 4A and 4B represent one patient treated with two fractions of SFRT in the same area, and cases 6A and 6B represent another patient treated with two fractions of SFRT in the same area. PD, Progressive disease; RD, Relapsed disease.

### Clinical and radiographic responses

**Figure 2:**
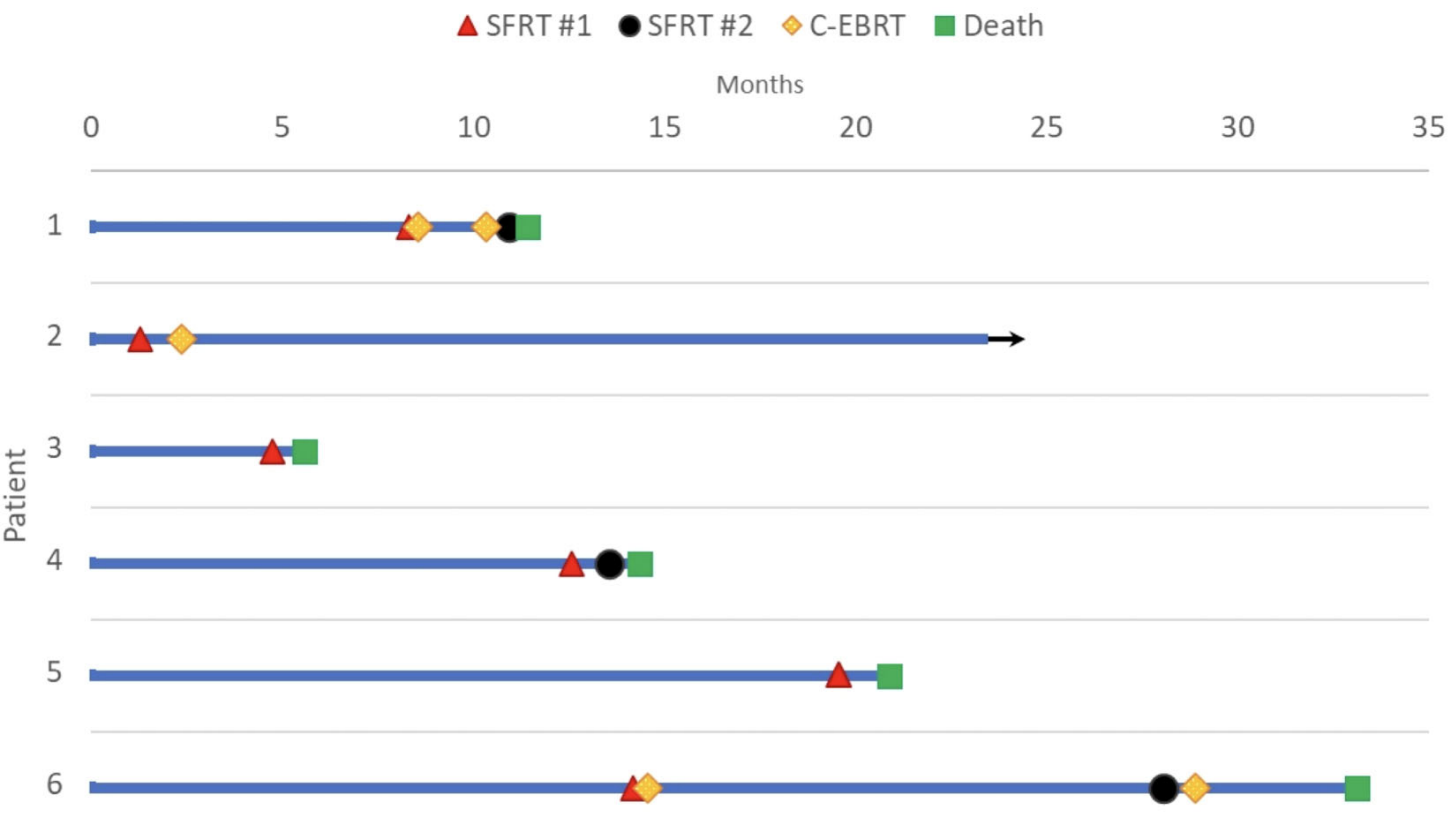
Swimmer plot of death and last follow up by month. Bar length indicates the duration of follow-up in months after the date of diagnosis. Red triangle marks the first SFRT course, black circle represents the second SFRT course, and green square represents death. Arrow represents continued follow-up for the surviving patient. Courses 1A and 1B are represented in Patient 1, Cases 4A and 4B are represented in Patient 4; Cases 6A and 6B are represented in Patient 6.

**Figure 3:**
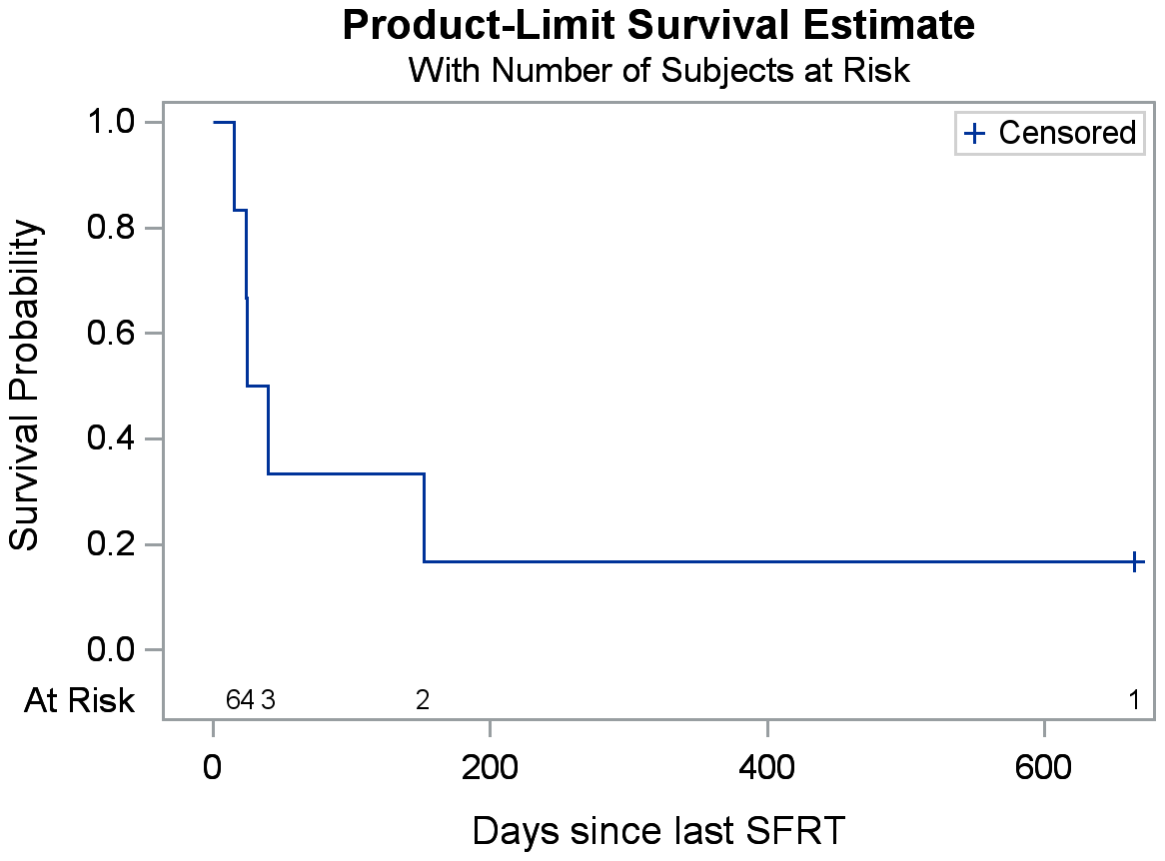
Kaplan-Meier estimates of overall survival after SFRT. (The total number of patients is six).

The median follow-up was 1.8 months (range: 0.5-21.9 months). Overall survival at the time of data collection was 17% (n=1). Among the treated sites, 67% (n=6) had follow-up imaging, revealing an average tumor volume reduction of 279.3 cc (34.2%), ranging from 32.8 cc to 1081.5 cc (7.5%-76.2%). Follow-up imaging could not be performed for three patients who passed away before their scheduled imaging appointments. Clinical treatment responses were observed in 66.7% (n=6) of treated tumors, including: pain relief (n=3), gastrointestinal symptom relief (e.g., decreased nausea/vomiting, improved appetite, n=4), AFP marker reduction from 169 to 101 ng/mL in a hepatocellular carcinoma patient 2.5 months post-SFRT, and improvement in biliary obstruction (evidenced by decrease in bilirubin from 39.9 to 21.3 mg/dL, resolution of jaundice, and normalization of stool color, 1 month post-SFRT). No grade 3+ toxicities were observed. **Table 2** provides a detailed summary of clinical responses following treatment, it shows clinical presentation, and radiographic and clinical responses of each patient’s courses after SFRT. A Swimmer’s Plot (**Figure 2**) shows a visual representation of overall survival, SFRT courses, and C-EBRT courses of these patients. A visual representation of survival (Kaplan-Meier survival estimation) was shown in **Figure 3**.

**Table 2:** Clinical presentation and responses for all SFRT cases.

Course #	SFRT peak dose (Gy)	Volume Before SFRT (cc)	Volume After SFRT (cc)	Clinical presentation	Clinical response	Follow-up c-EBRT
1A	15	1340	547	The patient presented initially with a large 15cm mass in retroperitoneum, found to have high risk neuroblastoma. Underwent initial resection and started chemotherapy on a clinical trial, but had disease progression, so started second line chemo and planned for first course of radiation.	One month after SFRT, there was improvement in appetite and clinical exam with midline mass less palpable. Chest X-Ray one month after SFRT showed improvement of pleural effusion.	5 Gy x 4
1B	15	445	412	Two months after Course 1A of SFRT, the patient had disease progression, with stability of irradiated tumors but overall progression of tumor burden in the hemithorax and pelvis. The patient initially underwent Quadshot, 14 Gy in 4 fractions, but there was increased growth in the hemithorax with increased mass effect on the left bronchi, so SFRT was given 16 days after Quadshot.	The patient expired 15 days after SFRT was delivered.	3.5 Gy x 4
2	15	797	247	The patient presented with a 12cm mass in iliopsoas muscle causing femoral nerve compression with enhancing osseous lesions in bone and pulmonary metastases. After the initial dose of SFRT, the patient completed a conventional plan to pre-operative dose of 42.75 Gy in 15 fractions to her sarcoma. Conventional radiation was complete 1 month after SFRT.	One week after SFRT, the patient’s pelvic pain was stable, but mobility improved, and the patient was able to walk with a walker. One month after SFRT, the pain improved. One year later, the patient was living a normal life and walking independently.	2.85 Gy x 15
3	15	266	N/A	The patient presented with a palpable abdominal mass and bilateral pulmonary nodules. The patient initially underwent left radical nephrectomy, chemotherapy, autologous transplant, then whole lung radiation. The patient relapsed 16 months after initial presentation with a 7cm mass in the left renal fossa, and underwent SFRT at that time.	One month after treatment, the patient presented to the ED with worsening pain and abdominal distension. The patient expired 25 days after completing SFRT.	No c-EBRT
4A	15	2306	1225	The patient presented with a liver mass and pulmonary nodules. The patient initially underwent chemo, then hepatic trisegmentectomy. After surgery, the patient enrolled in a CAR T-cell trial, with initial stabilization of clinical status and AFP. However, the patient progressed, so SFRT was offered. SFRT was preferred over whole liver radiation due to the patient’s poor liver function. The tumor involvement in the biliary system was so diffuse, there would be no appreciable benefit from surgery or interventional radiology.	The patient had one episode of acute grade 1 nausea and vomiting on the day of SFRT to the liver on the way home. Two months after SFRT, there was an improvement in grade 2 nausea, vomiting, and grade 2 pain, leading to a decreased need for pain medication, and improved ambulation. The abdomen became softer, swelling decreased, and stool color normalized from pale white to brown. Hyperbilirubinemia resolved as bilirubin decreased from 39.9 to 21.3 mg/dL over 1 month.	No c-EBRT
4B	10	1225	N/A	The first fraction of SFRT to the liver was used as a palliative measure for progressive disease. Since the patient improved clinically, a second fraction of SFRT was planned. The second fraction was done to the same target, the liver, but used a reduced dose of 10 Gy in 1 fraction (compared to 15 Gy in 1 fraction initially), and the high dose vertices were placed in different areas.	One week after SFRT, the patient had stable pain and slightly lighter stools. Three weeks after SFRT, the patient felt well enough to go on vacation. However, during this trip, the patient developed Klebsiella bacteremia, became hemodynamically unstable and subsequently expired.	No c-EBRT
5	10	270	N/A	The patient presented with a 15cm mass in the right kidney and pulmonary nodules. The patient underwent R nephrectomy and started systemic therapy. The patient first underwent whole lung radiation 12 Gy in 8 fractions, followed by a right flank boost of 7.5 Gy in 5 fractions. Then, the patient had a recurrent disease with spinal cord compression within the prior radiation fields. Therefore, an emergent plan of 4 Gy in 1 fraction was given initially, followed by a 10 Gy in 1 fraction SFRT plan, then continued conventional radiation to 12 Gy in 3 fractions. The total dose was 20 Gy in 5 fractions with a simultaneous integrated SFRT boost of 10Gy on the second fraction.	In the middle of the last R flank radiation fx 3/5, the patient was unable to move legs spontaneously or when encouraged to kick something. At the end of radiation, he had not regained strength in his legs. The patient traveled to another institution for a clinical trial but expired one month after completing SFRT.	4Gy x 4 fx
6A	15	598	142	The patient presented with a liver mass and initially underwent surgery. Chemotherapy was offered but the patient and family initially declined because of limited data for the efficacy of chemotherapy in this rare histology of hepatocellular carcinoma. However, the patient relapsed, so underwent chemotherapy at that point, with the addition of SFRT. The first course of SFRT was followed by 20 Gy in 5 fraction conventional radiation to the liver.	The patient had improved nausea/vomiting, less early satiety, and better PO intake after SFRT. AFP decreased from 9.6 to 5.6 ng/mL one month after SFRT.	4Gy x 5
6B	15	447	380	14 months after the initial course of SFRT, the patient had progressed through several lines of systemic therapy, and became more symptomatic from his disease, with increased pain to 7 out of 10. The second SFRT course was followed by 30 Gy in 10 fractions of conventional radiation to the liver.	There was an improvement in pain to 2 out of 10, six weeks after SFRT. AFP decreased from 169 to 101 ng/mL three months after SFRT.	3 Gy x 10

Cases 1A and 1B are for one patient treated with two courses of SFRT to two different anatomic locations. Cases 4A and 4B represent a patient treated with two fractions of SFRT in the same anatomic site, and cases 6A and 6B represent another patient treated with two fractions of SFRT in the same anatomic site. c-EBRT is the conventional unform field external beam radiation therapy.

### Dose metrics of SFRT plans

**Figure 4:**
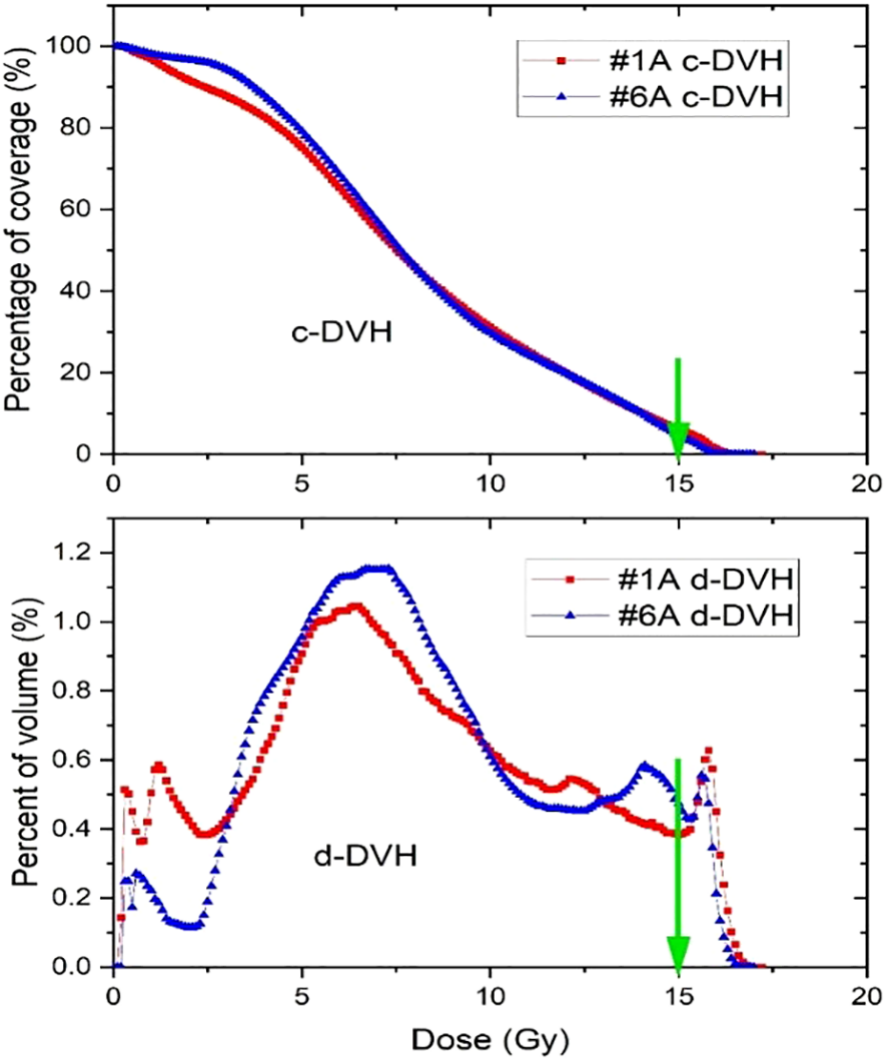
Cumulative and differential dose-volume histograms for two Lattice SFRT plans. The green arrows indicate the prescription dose.

Since a similar planning strategy was applied across all SFRT plans, the resulting dose distributions are relatively consistent. **Figure 4** illustrates two examples of DVH curves from separate SFRT plans. Though the patients had a large difference of target volumes (1340 *vs* 598 cc), a similar planning approach was used, so both plans showed similar DVH curves.

**Table 3** shows the dose and heterogeneous metrics of 9 SFRT courses. Due to the existence of ablative high dose cores which can effectively kill radioresistant cancer cells, a higher EUD was seen for a more radioresistant cancer. The D5/D95 ratio ranged from 2.23 to 11.42, with a mean value of 5.35 and a standard deviation of 2.87. In comparison, the D10/D90 ratio ranged from 2.04 to 5.99, with a mean of 3.68 and a standard deviation of 1.27. Using the same planning approach for all the treatment courses, D10/D90 demonstrates notably greater consistency as a dose heterogeneous index.

**Table 3:** Dose metrics (Gy) and D10/D90, D5/D95 ratios for 9 SFRT courses.

Course #	1A	1B	2	3	4A	4B	5	6A	6B
Prescription dose (Gy)	15	15	15	15	15	10	10	15	15
D100	0.70	0.30	0.20	1.35	0.30	2.50	0.30	0.30	0.60
D95	3.92	1.34	1.60	4.61	2.85	4.46	3.02	3.35	3.49
D90	4.55	2.42	2.32	5.35	3.73	4.75	3.60	4.10	4.40
D50	7.56	7.55	6.25	9.75	7.65	6.94	5.94	8.01	8.82
D10	14.08	14.50	13.43	14.76	14.01	9.71	10.10	14.72	14.55
D5	16.02	15.30	15.05	15.07	14.93	9.93	10.41	15.50	15.41
EUD (C1 Radiosensitive)	5.70	4.15	4.02	6.80	4.89	6.10	4.73	5.10	5.70
EUD (C2 Semi-sensitive)	6.46	5.24	4.86	7.72	5.92	6.48	5.34	6.20	6.74
EUD (C3 Radioresistant)	7.18	6.35	5.72	8.67	6.89	6.81	5.84	7.21	7.76
Heterogeneous index									
D10/D90	3.09	5.99	5.79	2.76	3.76	2.04	2.81	3.59	3.31
D5/D95	4.09	11.42	9.41	3.27	5.24	2.23	3.45	4.63	4.42

The unit for the prescription dose, D100, D95, D90, D50, D10, D5 is Gy. Case 1A/1B, 4A/4B, and 6A/6B represent three patients who received two fractions of LRT.

**Figure 5:**
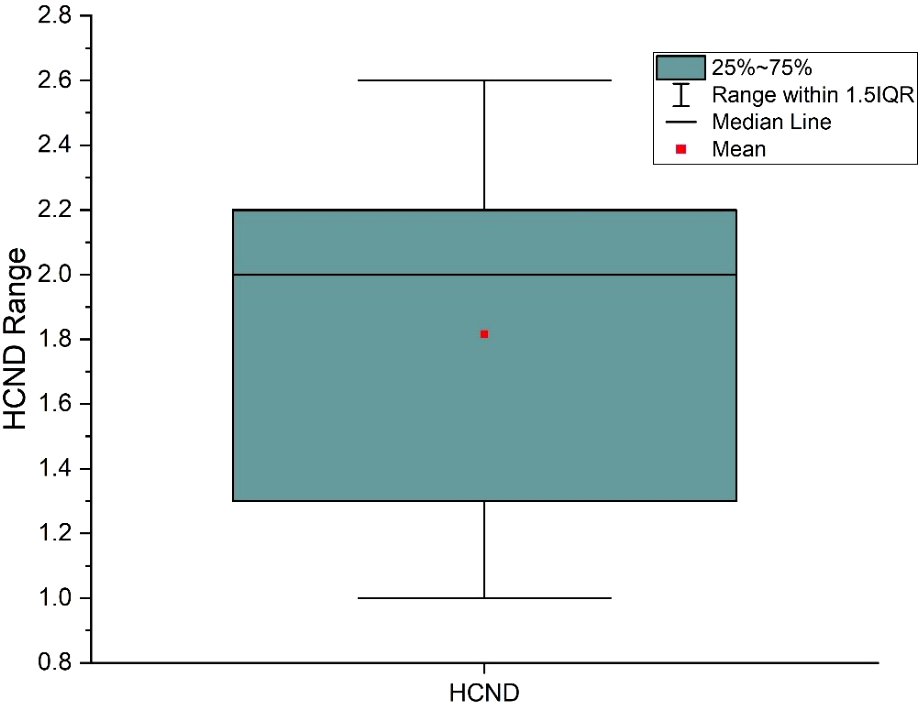
High dose core number density (HCND) variation range and mean value for the 9 SFRT courses.

From **Table 4**, across all cancer/normal tissue combination scenarios, radioresistant cancer cells (C3) consistently showed the highest TR, regardless of the radiosensitivity of the surrounding normal tissue. However, the maximum TR was observed when radioresistant cancer cells (C3) were interspersed within radiosensitive normal cells (N1) (C3+N1 scenario). The HCND ranged from 0.98 to 2.63 with the mean value of 1.82 and a standard deviation of 0.60. The consistency of HCND among all the courses was shown in **Figure 5**.

**Table 4:** Therapeutic ratio (TR) values for various courses of SFRT.

Course #	Number of high dose cores (n)	HCND	TR C1+N1	TR C2+N1	TR C3+N1	TR C1+N2	TR C2+N2	TR C3+N2	TR C1+N3	TR C2+N3	TR C3+N3
1A	18	1.34	1.48	1.26	11.47	0.71	1.26	2.30	0.61	0.83	1.12
1B	10	2.25	1.24	4.15	17.61	0.61	1.23	2.82	0.51	0.73	1.13
2	10	1.26	1.19	3.02	8.78	0.68	1.16	2.15	0.60	0.79	1.09
3	7	2.63	1.71	6.33	28.25	0.69	1.45	3.46	0.53	0.78	1.21
4-A	26	1.13	1.37	4.85	18.33	0.62	1.28	2.75	0.53	0.77	1.13
4-B	12	0.98	1.30	2.20	3.55	0.95	1.16	1.53	0.78	0.91	1.05
5	7	2.59	1.27	2.64	5.11	0.76	1.15	1.69	0.70	0.87	1.06
6-A	12	2.01	1.41	5.52	22.92	0.59	1.31	2.97	0.50	0.76	1.15
6-B	10	2.24	1.58	6.21	27.65	0.63	1.40	3.30	0.51	0.76	1.18

The highlighted columns indicate TRs of radioresistant cancer cells (C3) mixed with different normal cell types (N1, N2 and N3). Each treatment course was tested for nine different cancer-normal cell radiosensitivity combinations. Cases 1A/1B, 4A/4B, and 6A/6B represent three patients who received two fractions of LRT.

**Figure 6:**
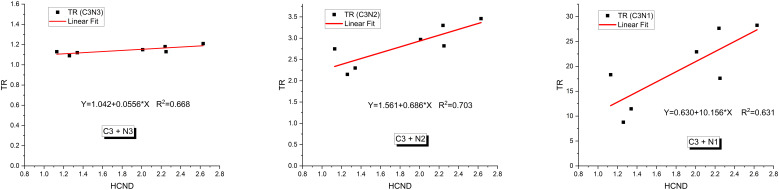
Relationships between the therapeutic ratio (TR) and high dose core number density (HCND) for radioresistant cancer cells (C3) interspersed in three types of normal cells (N1, N2 and N3) for the courses with prescribed dose of 15Gy.

The relationship between TR and HCND was analyzed in **Figure 6** using linear fitting. Although HCND varied between courses, in our limited cohort there was a strong correlation observed between TR and HCND (R² > 0.63), when the courses were tested at the same prescription dose (15 Gy). When treatment courses with different prescription doses were combined, the correlation between TR and HCND weakened (R² = 0.20). This is because TR is dependent on the prescription dose (31), whereas HCND is not.

## Discussions and conclusions

This retrospective study analyzed the outcomes of pediatric patients treated with SFRT for bulky tumors with palliative intent. A clinical response was observed in 66.7% of patients, with tumor volume reductions up to 76%. While SFRT has been established as a safe and effective treatment in adults, particularly for head and neck cancers, sarcomas, and gynecologic cancers (15–17, 21, 42), experience in the pediatric population remains limited. Implementing a prospective study of SFRT in pediatric patients presents challenges due to the heterogeneity of bulky tumors in this population, the scarcity of pediatric SFRT data, and the higher prevalence of bulky tumors in underserved populations, which are historically underrepresented in clinical trials (43). Tsang et al. (44) provides guidelines for palliation of pediatric tumors, suggesting 20 Gy in five fractions or 8 Gy in one fraction as viable radiation treatment options for metastatic disease in the liver, abdomen, and pelvis, however these approaches may be insufficient for bulky tumor palliation. As such, there is growing interest in alternative radiation techniques, including SBRT, proton therapy, and FLASH radiotherapy, for pediatric patients (44). To our knowledge, this is the first study reported in the literature assessing the use of SFRT exclusively in pediatric and young adult patients.

Despite the high radiosensitivity of many pediatric tumors, palliative radiation for pediatric oncology remains underutilized. Barriers include hesitancy towards radiation from parents, limited awareness among medical providers, and limited number of radiation oncologists experienced in pediatric care (45, 46). A study found that only 7.6% of 2202 pediatric oncology patients received palliative radiation at the end of life, despite 87% having a palliative care consultation (46). Another international retrospective study found that 83% of pediatric patients receiving palliative radiation for pain had a complete or partial pain response, leading to reduction or discontinuation of opioid medication in 46% of patients (47). These findings underscore the effectiveness of palliative radiation in pediatric patients, particularly for bulky tumors located near critical structures or in cases of re-irradiation.

Emerging evidence suggests SFRT may play a role in immune modulation. A study by Mohiuddin et al. demonstrated that SFRT using GRID therapy re-sensitized a pembrolizumab-refractory melanoma patient to immunotherapy, suggesting that high-dose GRID radiation therapy could act as an immune primer (48). Similarly, Jiang et al. (49) reported a complete response in one of the metastatic lung cancer lesions treated with LRT and concurrent anti-PD1 therapy, while other lesions treated with SBRT and CRT did not respond as effectively. Preclinical and clinical studies indicate that SFRT may create interspersed regions of intratumoral immune cell preservation and enhanced vascular perfusion, potentially improving anti-tumor immune activation (50). High-dose radiation at peak dose vertices may trigger antitumor immune responses by releasing tumor antigens and proinflammatory factors, promoting dendritic cell maturation and T-cell activation (51). Meanwhile, the low-dose valley regions may preserve tumor perfusion, allowing for sustained immune activation (52). Given that cancer cells exhibit poorer DNA repair capabilities than normal cells, the sublethal damage induced by low valley doses can preferentially kill cancer cells while sparing normal cells (30). Additionally, variations in dose distribution and spatial beam placement may enhance the consistency of abscopal responses, further contributing to the therapeutic effect of SFRT (53, 54).

Our radiobiological modeling offers insight into how cancer and normal cells, with varying radiosensitivities, respond to SFRT fields. The plan can be optimized if the radiosensitivities of both cancer and normal cells are known. The data showed that radioresistant cancer cells exhibit the highest EUDs with a significant TR (**Table 3**, **4**). Mixing radioresistant cancer cells with radiosensitive normal cells resulted in the highest TR. This occurs because the EUD for the lattice treatment is higher than the lattice valley dose which means for the same tumor cell kill, the radiosensitive normal cells get a lower dose with lattice than with a uniform dose treatment. The radiosensitive normal cells are better spared with lowered dose than the radioresistant normal cells, providing a better TR for lattice than a uniform dose treatment with the same tumor cell kill. This is consistent with findings from GRID therapy radiobiology modeling studies with cervical and melanoma cancer lines (30, 31). Although we are unable to identify which TR scenario in **Table 4** corresponds to each of our patients, ideally, the TR scenario for each patient would be determined before planning and this would drive the treatment planning process. This approach optimizes the therapeutic ratio even in the presence of cancer cells with varying radiosensitivities. In our limited cohort, TR demonstrated a dependence on high-dose core density (**Figure 5**). This indicates that increasing the number of high-dose cores may enhance TR, potentially allowing for a reduction in overall toxicity. Given its important radiobiological implications, this finding warrants further investigation in future studies.

### Limitations

Our study has several limitations, including the heterogeneity of tumor histologies and anatomic locations, and the small sample size and the variability in SFRT and C-EBRT dose/fractionation schedules. Our pediatric population also had low median overall survival, likely because SFRT was offered as a last-line palliative treatment option of last resort. Another limitation is the retrospective design of the study, which leads to variable clinical response assessments by the treating physicians and inconsistent follow-up schedules, introducing potential bias. Despite these limitations, our findings provide critical insights into the potential of SFRT for the palliation treatment of the pediatric population. Future prospective studies should aim to establish standardized SFRT dose, fractionation, and dosimetric planning protocols and implement structured follow-up schedules to assess clinical and radiographic outcomes consistently.

## Conclusion

Our results suggest that SFRT is a safe and effective palliative treatment or retreatment for pediatric patients with bulky tumors. This provides additional insights into an emerging treatment currently uncommonly offered to the pediatric population. In our preliminary study, SFRT exhibited high rates of symptomatic and radiographic response with a favorable toxicity profile, which supports SFRT as a promising treatment approach. Expanding research efforts in pediatric SFRT may ultimately expand this underutilized treatment option in pediatric oncology.

## Data Availability

The original contributions presented in the study are included in the article/**Supplementary Material**. Further inquiries can be directed to the corresponding authors.
