# Supplementary material for: Initial experience of spatially fractionated lattice radiation therapy for palliative treatment of pediatric bulky tumors
Source: Front Oncol. 2025 Oct 27;15:1648847. doi: 10.3389/fonc.2025.1648847 (PMC12597734; doi:10.3389/fonc.2025.1648847)
Supplement: Supplementary file 1 [file DataSheet1.docx]

# **Appendix A**

**Initial experience of spatially fractionated lactice radiation therapy for palliative treatment of pediatric bulky tumors**

**Note:**

This supplemental document provides information for understanding tumor locations, the theory and mathematical parameters used in radiobiological modeling.

1. **Supplementary Figure S1**

| **Patient 1**  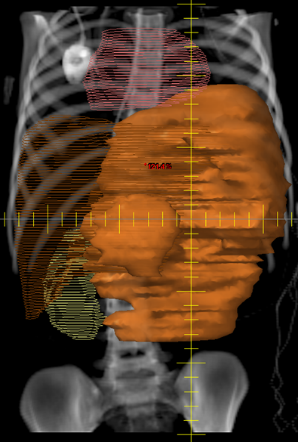 | **Patient 2**  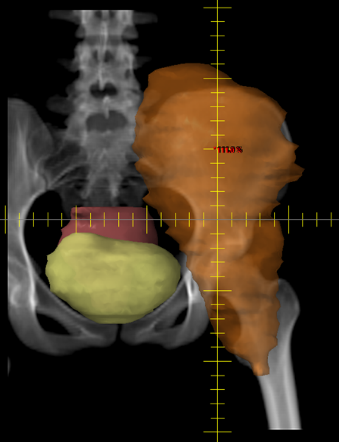 | **Patient 3**  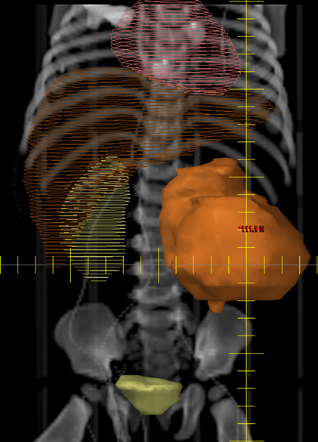 |
| --- | --- | --- |
| **Patient 4**  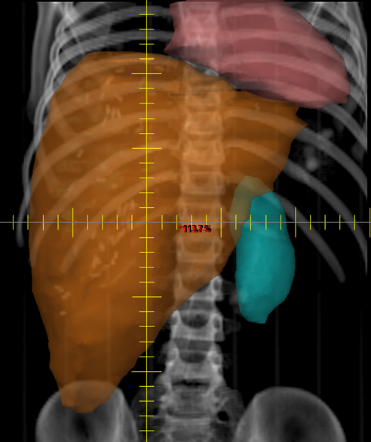 | **Patient 5**  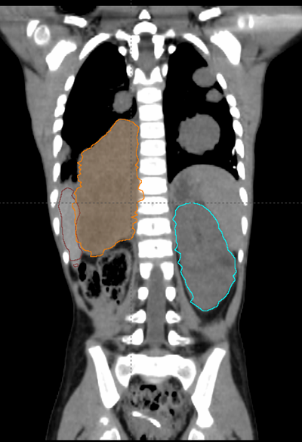 | **Patient 6**  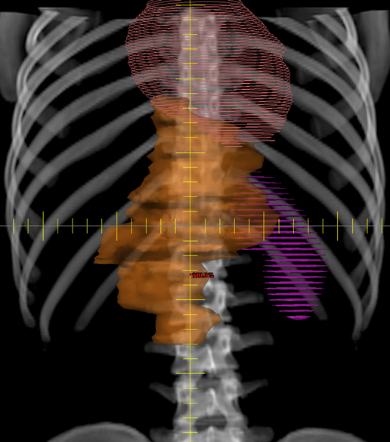 |

**Figure S1.** Contours for Initial SFRT Courses.

**Note:** The first SFRT Course is shown for patients 1, 4, and 6. The singular SFRT course is shown for patients 2, 3, and 5. CTV is orange.

1. **Supplementary description: A brief description of the radiobiology model theory and equations used in LRT.**

**Modified Linear Quadratic (MLQ) model**

The LQ model has traditionally been used to calculate tumor and normal cell survival fractions for a given dose of radiation ^34^. However, its accuracy for estimating survival at doses greater than 10 Gy has been questioned ^35,36^ in the past decade. To address this, the MLQ model, originally introduced by Guerrero and Li ^37^ for describing large dose radioresponses, was used to estimate post-radiation survival fractions of cell lines after LRT.

The MLQ survival fraction equation is expressed by the equation (1):

$SF(D_{i})=\exp(-\alpha\bullet D_{i}-\beta\bullet G(\lambda\bullet T+\delta\bullet D_{i})\bullet{D_{i}}^{2})$ (1)

Where *G(x) = 2(x + exp(-x) – 1)/(x)*^2^ is the dose protraction function, *λ* is the repair rate (λ=0.693/T_1/2_), and T_1/2_ is the repair half time (typically about 1 hour for most cells). *T* is the treatment delivery time, *SF(D_i_)* is the survival fraction at dose *D_i_*, and *α* and *β* are radiobiological parameters specific to the cell type. The dose protraction factor *δ∙D_i_* was included to correct for high dose killing*.*

In this study, δ was set to 0.15, T_1/2_ to 1 hour for all normal tissues and cancer cells, consistent with established MLQ model parameters ^38^. Variations in these individual values have a minimal impact on the survival fraction. However, the survival curve of the MLQ model becomes notably curved at higher dose ranges when considering all factors. Given that typical LRT treatment duration is approximately 15 minutes, a treatment time of T = 0.25 hour was used. The parameters used in the radiobiology modeling were summarized in Table A.

Using the differential dose-volume histogram (d-DVH) curve of each plan, and assuming that cancer cells are uniformly interspersed in the target volume (an assumption that may not be true for late-stage tumors), the average survival fraction, $\bar{SF}$for a given cancer cell radiosensitivity was calculated based on the MLQ equation and the sub-volumes of the tumor irradiated by corresponding doses. It is important to note that this averaging of cell survival assumes independent clonogens, excluding effects like bystander, abscopal, and cohort effects, which are hard to quantify and found more relevant to interspersed cancer cells than to normal tissues. ^35^

Using the average survival fraction, $\overline{SF}$, an EUD for a given treatment and cancer cell scenario (i.e. radiosensitivity) can be calculated by solving the MLQ equation ^32^.

When the above cell survival estimation approach is applied to the interspersed normal cells, a metric of therapeutic ratio (TR) can be calculated.^29^

$TR=\frac{{SF}_{Normal}(LRT)}{{SF}_{Normal}(EUD)}$ (2)

In Equation (2), SF_Normal_ (LRT) and SF_Normal_(EUD) are the normal cell survival fractions respectively in LRT and EUD treatments. A TR > 1 implies that a greater number of normal cells survive in LRT than in EBRT at the same rate of cancer cell killing, indicating a therapeutic advantage of LRT over traditional EBRT for sparing normal cells. ^31,32^

1. **Table A**. MLQ parameters for cancer cells and normal tissue used in the study ^38^*.*

| MLQ  parameters | Cancer cells (α/β=10 Gy) | | | Normal tissue cells (α/β=3.1 Gy) | | |
| --- | --- | --- | --- | --- | --- | --- |
|  | Radiosensitive  (C1) | moderate  Radiosensitive  (C2) | Radioresistant (C3) | Radiosensitive  (N1) | moderate  Radiosensitive  (N2) | Radioresistant (N3) |
| $\alpha$ (Gy^-1^) | 0.502 | 0.289 | 0.149 | 0.366 | 0.211 | 0.108 |
| β (Gy^-2^) | 0.0502 | 0.0289 | 0.0149 | 0.118 | 0.068 | 0.035 |
| δ | 0.15 | 0.15 | 0.15 | 0.15 | 0.15 | 0.15 |
| T_1/2_ (hour) | 1 | 1 | 1 | 1 | 1 | 1 |
| T (hour) | 0.25 | 0.25 | 0.25 | 0.25 | 0.25 | 0.25 |

**Note****:** The parameters can be applied in the MLQ (Modified Linear-Quadratic) model for radiobiologic modeling. However, if users choose to disregard the sublethal damage repair effect at high dose levels, these generic α and β values may be directly used within the framework of the traditional LQ (Linear-Quadratic) model.
